# Interesting Analyses of Articles in Foreign Journals

**Published:** 1864-11

**Authors:** 


### INTERESTING ANALYSES OF ARTICLES IN FOREIGN JOURNALS.

*Digitalis in Hæmoptysis.*—Speaking of hæmostatics in tubercular hæmoptysis, Dr. William Brinton says: "All else that I have to say of other remedies might almost be summed up in the statement, that it is in the terrible hœmorrhage of larger eroded vessels, and therefore of what is often advanced phthisis, that we have the most searching test of the value of any drug of this kind. Presuming we have such a means, we must very frequently use it. For it is often impossible to tell beforehand whether a slight oozing is not the commencement of a more considerable hœmorrhage, such as (directly or indirectly) endangers life. And I believe that we have such a remedy, for the majority of patients, in

*digitalis.* The ground for this strong personal conviction I find chiefly in the fact, that I have frequently seen profuse and alarming hœmoptysis, which had resisted many other means of treatment, including a judicious diet, and all the other drugs, yield to this active remedy, given so as to produce its well-known specific effects on the pulse. And not only so, but, in several cases, the interruption of the drug seems to have permitted the return of the hemorrhage, to be again arrested by the resumption of the digitalis."

*Carbolic Acid* (*Hydrated Oxide of Phenyle.*)—This substance, a product of the distillation of coal-tar, may now be obtained in any quantity, in a state of purity, and at a moderate price. As a chemical agent, it is a powerful antiseptic and antiferment, arresting putrefaction, the lactic fermentation, and the conversion of tannic acid into gallic acid and sugar. Experiments are now proceeding to ascertain whether it possesses the same power respecting alcoholic, butyric and acetous fermentation. On these grounds it has been recommended for experiment, as a remedy, and it has been used in several cases in the Manchester Royal Infirmary—by Dr. H. Browne in chronic diarrhœa; by Dr. Roberts in cases of vomiting and dyspepsia, attended with pain after eating; by Dr. Ransome, locally, for ulcers and offensive discharges; and by Dr. Thomas Turner in gangrenous sores and fetid discharges from diseased bone. In all cases it is alleged to be very beneficial. The dose is one drop in solution. For external application, one part of the acid to seven of water is the suitable proportion. Dr. Calvert states that it is a remedy for the foot-rot in sheep. Mr. Heath uses one part acid to two parts water to sloughing wounds.

*Exfoliation of the Female Bladder.*—Before the Obstetrical Society of London, Dr. Harley read his report upon Mr. Spencer Wells' case of exfoliation of the female bladder. The specimen was a bag, or sac, as large as a child's head, and perfect on all sides except one, where there were several rents. The exterior was of a white color, and distinctly muscular, the muscular fibres running in an interlacing manner, as in the urinary bladder. The interior was of a dark color, and everywhere covered with gritty deposit, on the removal of which a smooth mucous membrane came into view. The gritty deposit consisted of crystaline phosphates and urates. Over a limited portion of the external surface is a patch of a smooth membrane, like a serous membrane; and if such, was probably a portion of the peritoneum. No ureters nor orifices of ureters were to be found in the specimen, and the position of the urethra could not be ascertained in consequence of the rents in the lower portion of the specimen. For a similar reason, it was impossible to discover whether the specimen was an entire organ or only a portion of an organ. The question, "Was it a human bladder?" could not be easily answered on anatomical grounds, for in its present state the specimen had neither the size nor the shape of a human female bladder. The moral evidence, however, was all in favor of the view that it was, as has been described, an exfoliation of a human bladder. Dr. Tanner remarked that there was a preparation in the museum of the Royal College of Surgeons which might help to throw light upon the present specimen. It was the product of a man suffering from retention of urine from a fall, and in whom the catheter could not be introduced in consequence of striking against something like a membrane. He was relieved by the bladder being cut into above the pubis, which allowed a large quantity of purulent fluid and a membrane to escape. On examining the layer of membrane it is seen to be of sacular form, six inches long and four broad. Its shape indicates that it lined the whole interior of the bladder, and was thrown off in one piece. The outer surface is flocculent, and in parts distinctly fibrous; the inner surface is granular and reticulated, like superficially ulcerated mucous membrane. In fact, as the catalogue states, it exactly resembles the mucous membrane of the bladder, separated as a slough in one piece.

*Vaccination through Cow's Milk.*—M. Soubie, of Libourne, (France,) states, in the *Gazette des Hopitaux*, that he vaccinated the teats of a cow and obtained two fine vesicles. The milk of this cow was given to two children, one six months old and being brought up by hand, the other fourteen months and weaned.—The first took this milk for two days, on the fifth and sixth day of the vaccination of the cow; the second drank it only one day, on the eighth day of the vaccination. This latter child consumed about ten ounces of the milk; the first, nearly double that quantity. One month after this experiment the two children were vaccinated in the usual manner, but with a negative result; whilst the same lymph used with them acted very fully upon another child vaccinated at the same time. M. Soubie was induced to try this indirect mode of vaccination by a case in which a mother was suckling an infant, and who was attacked with small-pox, continued to nurse her child, the latter being affected with slight fever, without eruption. At two and five years this child was attempted to be vaccinated, without result; and even at sixteen vaccination proved of no effect. So M. Soubie inferred that that child had been protected by the milk of the mother.

*Œdema of the Larynx.*—Mr. M. W. Hilles says: "In this disease difficulty of breathing is induced by the elevated state of the mucous membrane lining the chordæ vocales, the epiglottis, &c., and is the result of the mechanical obstruction to the free passage of the air. Unfortunately, under these circumstances, a too small incision is made into the trachea or larynx, the effused fluid slowly exudes, the opening is obstructed, the difficulty of breathing continues, and the patient dies asphixiated. Now, the operation which I consider should be performed, is that of laying freely open the interior of the larynx; and the best mode of doing this is by division of the thyroid cartilage along the mesial line, the incision being carried downward through the cricoid cartilage, if necessary. By this means a free escape is given to the effused fluid, the breathing of the patient is provided for, and in a short time the elevated state of the mucous membrane will subside. I can see no rational objection to this operation; there is really no danger, and even the chordæ vocales need not be touched. In a few days the wound may be closed, and sufficient union of the alæ of the thyroid cartilage will be soon established. I had an opportunity of examining a patient who had attempted suicide by a longitudinal incision of the thyroid cartilage. The man nearly recovered, and the only symptom remaining was a husky state of the voice, which I have no doubt has since subsided."

*Reproduction of Bone.*—At a meeting of the Academy of Sciences, Dr. Lamare Piquot commuuicated a highly interesting paper relative to a case of regeneration of bone, which occurred in his practice. The patient, a boy of fourteen, met with a fearful railway accident, one of his arms being broken and one of his legs crushed under the wheels of a baggage truck. The tibia was smashed, or rather pulverized, into innumerable splinters, many of which were removed, and the fibula was in a like desperate condition. Although, from the nature of the case, a conservative course seemed to be rather hazardous than otherwise, nevertheless an attempt was made to save the leg. The limb was placed on a splint and subjected to continued irrigation for the space of thirtyseven days, the quantity of water daily used in the process varying from twenty to twenty-five quarts. At the end of three months the few remaining shreds of periosteum began to resume their bone-making functions; and so successfully is this second crop of osseous tissue proceeding at the present moment, that ere long the entire tibia will have been replaced by new material, and the boy be able to walk without crutches.

*Nævus Maternus; Deligation of the External and Common Carotids.*—M. Bertherand has communicated the following case to the Surgical Society of Paris. A child four months and a half old was brought to the Algiers Hospital with a nævus covering the whole of the left temporal fossa, reaching above to the vertex, posteriorly to the occipital protuberance, and below to and into the eyelids, which could not be opened. The extensive mole was the result of the rapid development of a merevascular stain at birth. M. Bertherand first tied the external carotid, whereupon the tumor diminished considerably, and success was thought complete; but within a few hours the vessels filled again. Perceiving this in the evening, M. Bertherand placed the child again under chloroform, and tied the common carotid artery. The diminution and sinking of the tumor were not so plain now as after the first operation. On the third day, however, it began to shrink, gradually withered, suppurated in some places, and finally disappeared. Not the slightest untoward sympton occurred. The author thinks this is the only case where so young a child (four months and a half) was successfully operated upon. He mentions Mr. Wardrop, whose patient underwent the operation at the age of six weeks, but died in consequence of it; M. Mayo, who operated at five months, but where a relapse took place; M. Wardrop, again, who tied the vessel at seven months, M. Rogers at eight, Pirogoff at nine, and Zeis at fifteen. Of these cases, the first two were successful, but the last two did not survive.

*Cutaneous Absorption.*—A paper on cutaneous absorption, by M. Parisot, was read at the Academy of Sciences by M. Claude Bernard. The conclusions which are established by the researches of M. Parisot are: 1st, that salts, such as iodide of potassium, chlorate of potash, yellow prusiate of potash, sulphate of iron, as well as the coloring matters of rhubarb, dissolved in water, are not in any way absorbed into the skin, even after two hours immersion, for not the slightest trace of their presence can be found either in the urine or in the saliva, by which they are generally eliminated, and in which they may usually be found, when they exist at all in the system. 2d, The poisonous vegetable alkaloids, digitaline and atropine, in solution are likewise not absorbed into the skin, for a prolonged sojourn in a bath containing a strong dose of these substances, does not give rise to any symptoms of poisoning.

*The Dyalizer.*—The chief merit of the "dyalizer," or apparatus for effecting analysis by diffusion, is its extreme simplictity. It consists of nothing more than a species of seive, having gutta-percha sides and a parchment bottom. When in use, it is floated in a basin of distilled water, and the substances under examination are thrown on it as on a filter, and then allowed to remain undisturbed for twenty-four hours. At the end of this time all the crystallizable salts will be found to have passed through the vegetable parchment bottom, while the organic matter is left behind. By this means, arsenious and metallic salts, strychnine, and other poisons, can be readily separated from organic solutions in medico-legal inquiries, without much loss of time, and entirely without much trouble.

*An Englishman on French Hospital Treatment.*—An old coachman, now of any age above eighty, a regular John Bull, sent me a pressing message one day to call in and see him, as he considered himself in a dying state. "What is the matter, George?" said I, on entering. "Oh, you may well ask what. Everything's the matter. I'm blind and deaf and dumb, and hard-by dead.—They've done it for me, they have." "Who?" said I. "Oh, them chaps, them d—d French hockilix (oculists?). I've been in a hospital, sir, with a strong weakness, sir, in this here heye, sir, as is hout, sir; and if you'd knowed, sir, the messes I have been made to eat, sir, you would'nt have asked 'what,' I'm thinking. A morning, sir, soup—greasy water it was; eleven o'clock, beef—rags, sir, rags; evening, sir, more soup, more like in the morning. And if that then for seven long months aint enough to do for an English, with insides as is English, why then I don't know a horse from a donkey, sir." "Had you no wine?" replied I. "Oh, yes, sir, plenty of wine, plenty, and winegar was a joke to it. Oh, yes, plenty of wine, plenty;" and here my patient went into a melancholy chuckle. I believe poor old George had, in his own personal experience, stumbled against one of the real causes of the increased rate of mortality in the Paris hospitals.—*Lancet.*

*Cause of Pyæmia.*—In a discussion before the Medical Society of London, Mr. Henry Lee stated that of all the blood-poisons, none was so deleterious as decomposed fibrin. It will, he said, produce much more formidable symptoms than the introduction of any amount of healthy pus; and he believed secondary abscesses and typhoid symptoms were to be referred to this cause. Dr. Richardson thought that in cases of purulent inflammation it was a question, first, whether there was a preliminary stage in which pus was formed in an organ or tissue previous to the blood-poisoning, or, secondly, whether the change took place primarily in the blood? He did not believe in the absorption of pus, and considered that the change originated from within, and not from without, and that pus formed outside the circulation did not enter into the blood. The mere injection of healthy pus into the blood will not produce blood-poisoning. One drachm of pus, when thrown into the circulation, will not often affect a dog. He thought that in all probability pus was formed out of fibrin, and that the formation of abscesses was beneficial. Mr. Barwell remarked, that he did not believe that absorption of pus was a cause of purulent inflammation. A physiological society in Edinburgh had injected four ounces of healthy pus into the jugular vein of a donkey, and the animal recovered. The operation was repeated in a few months afterwards without any injurious results following.

*Liquid Permanganate of Potash.*—M. Leconte prepares this solution in the following manner: Caustic potash, six drachms; chlorate of potash, five drachms; binoxide of manganese, five drachms. Dissolve the caustic potash and the chlorate in a small quantity of water, and add the manganese; get rid of the water by evaporation, stirring constantly, and calcine the dry mass to a dark red for an hour in an untinned iron cup; allow to cool, and add a quart of plain water. Then boil for five minutes in a china capsule, and you will obtain a fluid of a slightly purplish tint; decant the solution, and wash the residue with such a quantity of water as to make altogether two quarts.—When filtering is thought necessary, the liquid should be passed, not through paper, but through very fine sand. For dressing foul wounds, or for injecting, use one drachm of this solution to from three drachms to five of spring water.

### *Case of Popliteal Aneurism cured by Digital Pressure.* By George Southaur, F. R. C. S.

The patient was in his thirty-third year, an iron-moulder by trade. From the man's account the disease did not appear to have been of more than nine or ten weeks' duration. The right popliteal space was distended by a pulsatile swelling, accompanied by severe pain and general œdema of the leg. The pulsation of the tumor was very perceptible, but feeble, and the skin over it slightly discolored. His countenance bore indications of severe suffering. Pulse 120, small and quick; appetite bad. So urgent did the case appear, that clamps were immediately applied over the femoral artery. The limb was enveloped in flannel bandages, and elevated on pillows; bottles of hot water were placed near, to raise the temperature of the foot and leg to the natural standard. The following day the clamps were removed, as they had produced redness and vesication of the skin. Iodide of potassium was now prescribed, and regularly taken for three weeks, with no apparent improvement. Digital compression was then resorted to, twelve students having volunteered their services. During the first twenty hours this system of pressure was frequently interrupted, and therefore at the end of this period the pulsation had apparently undergone no change. Consequently two students were directed to be continually with the patient, one to compress the artery, the other to apply his hand over the aneurism to detect any insufficiency of the pressure. This plan was adopted for twenty-four hours; a very slight pulsation could then be felt in the tumor, and six hours later it had entirely subsided. Moderate pressure was, however, kept up for another day, and then discontinued, as there were no signs of any further pulsation. From this time the case proceeded very satisfactorily, the man leaving this hospital at the end of three months, with scarcely any remaining traces of the disease.

Mr. Southaur considers that pressure must now be regarded as the established system of treating aneurismal tumors whenever practicable; but the best mode of applying it is still a subject for discussion. In the present case the vitality of the limb was evidently too much impaired to admit of instrumental pressure; indeed, the unusual size of the tumor, the unsatisfactory condition of the surrounding parts, as well as the patient's general health, formed in themselves serious obstacles to any kind of operative interference. Many are the advantages of digital over instrumental compression. Not only does it seem to effect a cure in a shorter period of time, but with much less pain, and is not so likely to lead to sloughing of the structures under pressure, which with the greatest precaution is liable to occur in some cases. The flow of blood through the aneurism may not be sufficient by this means, and indeed this is not to be desired, as fibrillation of the blood is more likely to occur, and to be more permanent, if allowed to pass through the sac in small quantity and in a slow continuous stream; for digital compression is not liable to those sudden alternations in force and volume, which under instrumental pressure are apt to take place, in consequence of the tendency of the artery to escape from under the clamps on any slight movement of the limb. But pressure alone must not be entirely relied on in the treatment of aneurism; for as success depends on the consolidation and subsequent absorption of the blood in the tumor, other agents of similar properties, to combine with, must be sought for. The difficulty in arriving at correct views of the medical treatment of aneurism is probably owing to our imperfect knowledge of the changes which contribute to the solidification of the aneurismal contents.

The process is usually regarded as similar to the coagulation of the blood out of the body; but it is questionable if this be exactly so where the solidified material is partially absorbed or converted into structure. Ordinary coagulation of the blood is one consequence of its diminished vitality, and therefore would more likely be followed by its removal from the body by ulceration and suppuration than by absorption. It seems, therefore, highly probable that the solidification of the blood within the body, previous to its absorption or conversion into tissue, differs somewhat at least from that of ordinary coagulation. This may explain how it is that low diet, which, by depressing the vital properties of the blood, should promote its coagulation, has not led to those salutary results expected by its advocates in the treatment of aneurism.

### *Treatment of Strabismus in the Royal Ophthalmic Hospital.* By George Lawson, Esq, F. R. C. S.

Much has been lately said and written on the treatment of strabismus, and different surgeons have laid claim to special operations for the remedy of this defect, urging that the method they suggest does away with many of the objections which are attributed to the subconjunctival operation.

Before, however, suggesting improvements upon the mode of performing an operation, it would be well if the author of every new method had a right appreciation of the proper way of performing the operation he seeks to supersede.

It has been stated and printed in a public journal, that some of the objections to the subconjunctival operation are, that the plica semilunaris is interfered with, and that the result is a shrinking and retraction of the caruncle. Again, it is urged that suppuration and large growths of granulations follow the operation, and add considerably to the after deformity.

To such statements I can only say that the writer of them either could never have seen the subconjunctival operation properly performed, or else must have witnessed results very different from those which are obtained at the hospital.

I would preface the remarks I have to make on this operation by stating that the plica semilunaris ought never to be interfered with; that the falling back of the caruncle is an exceedingly rare occurrence after the operation, and cannot follow unless improperly performed; and that suppuration of the wound and the after-formation of granulations in the site of the cut in the conjunctiva never occur.

I will now briefly describe the operation as described at the hospital.

The lids are kept apart by the ordinary wire speculum. The surgeon then makes a small opening in the conjunctiva with scissors, over the lower edge of the insertion of the rectus tendon, taking hold of the membrane, and often the deep fascia at the same time, with the forceps, which, if the eye be turned inwards, may be slided (closed) along the surface from the edge of the cornea till they reach the proper spot for the opening: thus the eye need not be held by an assistant. The fascia being opened, the lower edge of the tendon is exposed *close to its insertion.* If the fascia has not been opened at the first snip, it is in turn seized by the forceps at the same point and divided, without interference with any other structure; the object being simply to divide the tendon on the ocular side of the hook *at its insertion.* The blunt hook is now passed through the aperture in the subconjunctival fascia, and behind the tendon, which it renders tense by being made to draw on it slightly forwards and outwards. The next step is the introduction of the scissors. Mr. Bowman insists on the propriety of carefully introducing the points of the scissors, not much separated—one along the hook behind the tendon, the other in front of the tendon, and between it and the conjunctiva, and of dividing the tendon by *successive snips* from the lower to the upper edge. If the tendon is divided by one cut, the operation is more roughly executed, for, as the blades have to be opened more widely, the opening in the conjunctiva and fascia must be larger, vessels of a larger size may be divided, and the tendon may be pushed off the hook before the points of the scissors; if this happen, of course the hook must be re-introduced. The surgeon completes the operation by making a small counter-puncture, by bulging the conjunctiva on the end of the hook in the situation of the upper border of the tendon after its division, and by then snipping it with the scissors; the object being to allow any of the effused blood immediately to escape, instead of diffusing itself over the sclerotic. The subsequent ecchymosis then never need extend beyond the seat of the operation, and should disappear within a few days.

The results of this operation, when properly performed, are so satisfactory that I feel any new method must possess very strong claims to justify its preference.

Before operating upon a patient for strabismus, the visual condition of the two eyes is to be ascertained, and the relative strength of the internal and external recti muscles made out. Mr. Bowman is very decided in urging the necessity of carefully estimating the comparative strength of these muscles in both eyes, as according to their relative power he determines upon the necessity of operating upon one or both eyes. He has adopted a set of symbols which indicate accurately their comparative state. The patient is made to look at a near object held at the extreme outer limit of his field of vision, first on one side, then on the other, and the extreme limit of movement of each eye inwards and outwards is then noted, with reference respectively to the lower punctum and the outer canthus; the pupil being the part of the eye used to mark the movement inwards; the outer edge of the cornea the movement outwards. In noting the case on paper, the diagrams of the positions of the two eyes should be placed on the same line, as if facing the observer; that of the right eye on the left hand side.

In each case the exact distance admits of being recorded. In this manner the relative strength of the internal and external recti may be estimated, and the result marked down in a single line, so as to show at a glance in which eye the preponderance of power of either muscle exists. After the operation another examination is made, and the result again marked down. We are thus enabled accurately to record on paper the amount of power the one muscle has gained and the loss the other has sustained by the operation, and this at successive periods in the history of each case.

*Carbonic Acid in the Atmosphere.*—At a sitting of the French Academy of Sciences, M. Mine read a paper on the quantity of carbonic acid in the air, showing that it does not exist in the same amount throughout the year. During December and January the quantity remains nearly the same; it increases in February, March, April and May, and diminishes during the months of June, July and August; after which it increases again from September to November, the maximum of the whole year occurring in October. During the night it is more abundant than in the day time. The maximum quantity during the day occurs at noon. After a shower it always experiences an increase.

*Ergot of Wheat.*—M. Leperdriel has proposed the employment of the ergot of wheat as a substitute for that of rye. The reasons for which, as assigned by him, are: 1st. It does not undergo decay or change very quickly. 2d. It contains 15 per cent. less of the poisonous resinous principle, and 20 per cent. more of the efficacious principle of the ergots.

*Treatment of Malarious Fever by the Subcutaneous Injection of Quinine.*—By W. J. Moore, of the Bombay Medical Service.

Since the year 1858, when Dr. Wood brought forward the hypodermic method of administering morphia, the plan has been extensively tried. Moreover, the results following the injection of morphia into the subcutaneous areolar tissue have, on the whole, been satisfactory, and the use of the alkaloid in this manner has now become an established practice in various obstinate neuralgic disorders. Other agents, as atropia, have also been used hypodermically with varied success, and I have latterly employed a strong solution of quinine for the cure of intermittent and remittent fever by the method of subcutaneous injection.

The success which has attended the practice renders me desirous of calling attention to this novel mode of using quinine. I have so employed the remedy in upwards of thirty cases of intermittent fever, and in several cases of remittent, and with almost invariable success; the former class seldom requiring a second application, the latter generally subsiding after the fifth or sixth injection. Since the period I commenced to use quinine in this manner, I have been surprised and pleased to find in one of the medical periodicals, that the same plan has been pursued by Dr. Chasseaud, of Smyrna, who reports one hundred and fifty cures, and especially recommends the system in fever, complicated with gastric symptoms, when the exhibition of quinine by the mouth is often "inefficient, difficult and hazardous."

I use the strongest solution of quinine which can be prepared—viz: thirty grains of quinine, eight or ten drops of dilute sulphuric acid, and half an ounce of water. Of this I inject from half a drachm to a drachm, the former quantity containing some four grains of the active agent. With the exception of a little sulphate of soda, if the bowels are confined, I use no other remedy whatever in uncomplicated cases of any type of malarious fever. When the spleen is enlarged, or if a leucocythemic condition is present, I prescribe, as an additional curative agent, one or other of the preparations of iron—very frequently the citrate of iron and quinine.

I generally inject beneath the skin, over the outer belly of the triceps extensor muscle, and sometimes over the deltoid. I have, however, used the syringe with equal effect on the thigh and calf, and, in cases of enlarged spleen, have thought the action of the remedy increased by injecting over that organ. I use a small glass syringe, with the screw action, and furnished with a sharp silver point, some half an inch in length. The latter is introduced beneath the integument half an inch or less, and the pain is not greater than the prick of a pin. Indeed, patients have frequently declared that they would rather submit to this process than taste the bitter of quinine. I have never seen the slightest inflammation or irritation follow the operation except in two instances. In one of these the result was due to the instruments employed—namely, a small trocar and common glass syringe; in the other, to quinine in *suspension* being used, instead of in *solution*. Indeed, I have reason to believe that quinine in suspension is very irritating to the tissues, and this is what physiology would lead us to expect, as it is certain that when a fluid material is introduced into the areolar structure, it will be absorbed more readily than any solid mass could be. Therefore, to avoid irritation of the parts, and, also to prevent "choking," I insist upon a perfectly clear solution of the alkaloid.

The best time to inject is shortly before the expected cold fit, but it may be done during the first stage with the effect of lessening, and sometimes stopping the whole paroxysm. Latterly, when a patient presents at the morning visit, who expects an accession during the day, I have injected at the time, and nearly invariably the fever has stopped.

In cases of remittent, I have endeavored to inject during the remission, but do not wait for this period. In severe cases the injection should be repeated at intervals of six or eight hours.

I believe four or five grains of quinine injected beneath the integument are equal in their effects to five or six times that amount taken into the stomach; also, that the effects are more certain than when taken in the ordinary method; and, also, that relapsing attacks are less common than when the remedy is administered by the mouth.

*On Fractures*—By W. H. B. Winchester, F. R. C S.

There is no subject within the entire range of surgery on which so much has been written and yet so little real practical improvement made as that of fractures. A system so long and perseveringly followed as that on which the present treatment is based, but which has proved so barren of good results, may reasonably be suspected of embracing some radical defect both of principle and practice. It will not be amiss, therefore, to examine one or two of its leading features, for the purpose of discovering the cause and its remedy.

In the first place, with regard to Extension. The views entertained respecting it are so erroneous, that until they are entirely abandoned, no improvement can take place. Muscular contraction has been, and still is, the real bugbear. To counteract its supposed baneful influence, or, in other words, to keep up permanent extension, has been the chief object sought to be attained, and no combination of forces the ingenuity of mechanicians could devise, has been considered too powerful to attempt its accomplishment. Look, for instance, at the powerful screws attached to the extremities of some instruments—at the railway splint of Professor Dumreicher, some time since used in St. Bartholomew's Hospital, by which the lower part of the splint, supporting and holding in its grasp the lower or separated portion of the limb, is so contrived as to run away with it to the utmost limit of muscular elasticity. Such, too, is the effect of attaching a heavy weight to a cord from the foot, and letting it run over a pulley at the end of the bedstead. A similar result also follows the application of the most commonly used of all splints—viz: Desault's long splint, and of all ordinary forms of apparatus. Extension, therefore, as at present understood and practised by the above means, is a persistently active, and as such an injurious, force. It can only be beneficial when temporarily employed to effect replacement. As soon as this is accomplished it ought to be superseded by the passive, enduring, and consequently beneficial force expressed by the term Retention.

Now, this much-dreaded muscular contraction, the involuntary result of fracture, hitherto considered so obstructive, discarded as even worse than useless, and opposed as injurious, is, if rightly understood, a natural power of inestimable value, supplying the exact amount of forcible contact between the broken surfaces necessary to excite healthy reparative action in the most speedy and perfect manner, accurately adjusted to the functional capacity of each individual case. That this view is correct, physiologically, pathologically, and mechanically, will undoubtedly be admitted. At least nine-tenths of the cases of ununited fractures, of tardy union and deformity, may be attributed to the misunderstanding, neglect, or overlooking of this obvious pathological effect of muscular contraction.

Paget, in the last edition of his admirable treatise on Surgical Pathology, when speaking of ununited fractures, (page 193,) remarks: "In other cases the failure seems to occur earlier. No reparative material is formed, and the fragments remain quite disunited. This may be the result of accidental hindrances of the normal reparative process; but it sometimes appears like a simple defect of formative power—a defect which, I believe, cannot be explained, and which seems the more remarkable when we observe the many changes which may at a later time be effected, as if to diminish the evil of want of union." Is not this its true solution or explanation—viz: that nature, not uneqal to the task, has only been deprived by art of the very means which she has specially provided for its accomplishment? How is this provision of nature to be rendered available? By adopting the means I have frequently had occasion to describe—viz: the necessity of aiding and directing nature by suitable, and not retarding or obstructing her by improper forms of apparatus.

*Case of Cirrhosis of the Liver in a Child Five Years Old.*—M. C——, aged five years, came under Dr. Murray's care about three weeks before her death, complaining of severe pains in the abdomen, with bearing down, and passage of blood. On investigating her history, it was found that up to three years of age she had always been a very healthy child, when at this time she began to experience transitory pains in the abdomen, more especially in the hepatic region. At times these were so severe that the child could not tolerate them, and, though naturally patient, she screamed in agony. Soon afterwards she began to be affected with a sensation of bearing down, which caused her to go to stool very frequently. Her constitution began to give way, and from being strong and healthy she became pale and anæmic. The mother stated that she applied to several medical men, without, however, deriving any good. At this time no suspicion was entertained regarding the nature of the case. She continued in this chronic condition of ill-health, having the bearing down sensation and pain generally diffused over the abdomen, when the mother's attention was attracted by the child's passing blood at stool. She was then placed under Dr. Murray's care. On examination, the abdomen was found to be very much distended, but no fluctuation could be detected. The liver was hard and nodulated to the touch, and when pressed upon the child cried out; the feeling was exactly similar to that presented by a cirrhosed liver. To relieve the pain, warm fomentations were applied over the abdomen, and a few doses of Dover's powder given, which had the desired effect. About a fortnight after she was first seen the patient was attacked with severe hæmatemesis, when she vomited several pints of dark venous-looking clotted blood. A few doses of gallic acid, with cold cloths, and rest in the recumbent posture were ordered, which, for the time, had the effect of stopping the hemorrhage; but a few hours afterwards a second attack supervened, which resulted in the death of the child.

As a wish was expressed to have a post-mortem examination, the mother willingly complied with it. On making an incision through the abdominal parieties, the first object which presented itself was the liver, which was covered with nodules, varying in size from a hazel nut to a hen's egg. The parenchyma presented everywhere a granular character, and of a dense, firm, leathery consistence. The granulations varied in size from a pin's head to a linseed, and were separated by corresponding areolar tissue. The intestines were universally covered with ecchymosed spots, and from the rectum upwards were filled with masses and clots of dark venous-looking blood. The stomach also was packed full of clots. The whole surface of the mucous membrane, from the stomach downwards, was carefully washed and examined, but no lesion of any kind could be detected; thus proving that the blood was entirely passive in its origin, arising from the obstruction to the passage of the blood through the portal vein. The spleen was greatly enlarged and much congested. No examination was made of the head.

Here was a case of distinct cirrhosis of the liver in a child only five years of age, which was not congenital, for she enjoyed good health till she was three years old, nor could any particular cause be assigned for it. The child herself had a peculiar repugnance to alcoholic stimulants of every kind. With regard to the parents themselves, they certainly were very intemperate in their habits. The records of children's complaints do not present a similar case in so young an individual.

*On the Employment of the Alkaloid of the Calabar Bean in Prolapsus of the Iris.*—Mr. Thomas Nunnelly calls attention to the great advantage we may derive by availing ourselves of the undoubted power of the Calabar bean over the concentric fibres of the iris, by which, in the course of a few minutes, the pupil may be reduced in size to a mere speck, and the whole surface of the iris put upon the stretch; the direction of the force being from the circumference towards the centre of the membrane, precisely as is desired in the cases referred to.

There are few surgeons who do not know how frequently the cornea and anterior margin of the sclerotic are opened by punctured and incised wounds: in women and children by the point of a knife, scissors, knitting-needle, steel pen, and similar instruments; in men by the flying off of a splinter of metal, stone, or other hard brittle substance, in the varied mechanic works going on all over the country. And every surgeon also knows how unsatisfactory, in the result, most commonly such wounds are, occasioning great pain and distress, often tedious confinement and loss of health, and, at the best, in the great majority of cases, resulting in permanent impairment of the eye, more or less considerable, according to the extent of the prolapsus of the iris and the severity of the inflammation occasioned by it: a *perfect* cure being the exception rather than the rule.

Apart, however, from the protrusion of the iris, which almost invariably occurs whenever the cornea or the margin of the sclerotic is accidentally wounded, there is really no reason why these wounds should be so troublesome. Under favorable circumstances, wounds of these tissues heal rapidly. Punctured wounds of the cornea and sclerotic, when made by the surgeon, almost invariably heal at once, and not unfrequently the large corneal incision made in extraction of the lens is healed in twenty-four hours. Now, as in the great majority of cases the instrument by which these accidental wounds are inflected is small and sharp, these are incised wounds in their character; and thus there does not appear to be any reason why, if the iris could only be kept from prolapsing, they should not heal as readily. Of course, where, as sometimes happens, the offending substance is large and the impelling force considerable, the coats of the eye are lacerated, and the whole ball is contused; but, even in these cases, if the lens be neither wounded nor displaced, so highly organized is the ball, and so great its reparative power, that I am convinced, could the iris only be kept in its normal situation, and the lips of the wound in the cornea and sclerotic be kept in contact, many eyes which are now lost would be saved, and the patients escape great and prolonged suffering.

Many plans have been suggested for disengaging the prolapsed iris, which, though occasionally successful, far more commonly fail. It occurred to me that if the iris could be kept for some hours on the full stretch, by the almost entire contraction of the pupil, it would not prolapse, and thus the corneal wound might heal by the first intention. The result of two cases in which I have employed the alkaloid (for such I presume it to be) of the Calabar bean is most satisfactory, and would quite justify the belief that if the case be seen immediately after the infliction of the injury, before prolapsus has taken place, or, even though this has happened, before adhesion has occurred, the iris may be kept out of the wound, and this will then heal as after a surgical wound.

*Poisonous Snake Bites.*—Dr. Shaw, of New South Wales, states that a short time since he was requested to visit a young man who had been bitten on the arm by a black snake, one of the most deadly of the Australian varieties. As the patient lived a few miles from his residence, it was nearly two hours from the time of the accident till he saw him. Upon arriving, he found him in a very low condition; his countenance very pale and listless; body bedewed with a cold perspiration; the pulse small, rapid and fluttering, with great drowsiness and disinclination to speak or to answer questions. A ligature had been applied by a neighbor, above the wound, shortly after the injury. The doctor seized the bitten part with forceps, and cleanly excised it and around it to the extent in size of a shilling. Pretty free bleeding occurred, which was further encouraged by getting the lad's father to suck the wound with the mouth. The sucking being continued for about ten minutes, he then applied a strong solution of ammonia to the wound, and, at the same time, gave a draught, consisting of two drachms of aromatic spirit of ammonia, and the same quantity of tincture of assafœtida in a little water, and this he ordered to be repeated every hour, with strong coffee *ad libitum.* Soon after these measures were adopted, and especially after the administration of the draught, he appeared to revive, and continued to do so well, that in three hours from the time of the visit he was considered completely out of danger.

In the treatment of this case, no originality is claimed, with the exception of the administration of tincture of assafœtida, and for that hint he was indebted to Dr. Frances Cambell, of N. S. W., Dr. C. appears to look upon the tincture of assafœtida as almost a specific in snake bites, and certainly the result of cases he adduces which were treated by that medicine would almost warrant one to endorse his opinion. The probability is, however, that it owes its properties in such cases more to its action as a stimulant, and perhaps, also in part to its bad taste and odour, rousing, to a certain extent, the depressed nervous energy of the sufferers from that species of poison.

Dr. Berncastle, of Sydney, another gentleman who has paid particular attention to snake bites, considers stimulants the great *forte* in the treatment of such cases; and although he advocates the propriety of local treatment, still he appears to say that it is of less importance than the administration of large doses of some diffusible stimulant, he giving the preference to whiskey.

The rational course of treatment, then, appears to be to get rid of the poison by excision or free scarification of the wounded part, and by propping up the depressed powers of life by diffusible stimulants of any kind till the shock—always an attendant of snake bites—is recovered from.

*Rare Case of Tetanus.*—A boy died at St. Bartholomew's Hospital from slight injuries and a scratch received on the nose, terminating in tetanus. The injuries were caused by the attempt being made to strike him with a board, which unfortunately caught his nose and grazed it. The wound went on very well for a while, he became worse, all the symptoms of tetanus set in, and he died.

*Effects of Tobacco.*—In a paper read before the National Association for the Promotion of Social Science, Dr. Richardson comes to the following conclusions, in which every position has been founded upon individual research:

1. The effects that result from smoking are due to different agents imbibed by the smoker—viz: carbonic acid, ammonia, nicotine, a volatile empyreumatic substance, and a bitter extract. The more common effects are traceable to the carbonic acid and ammonia; the rarer and more severe to the nicotine, the empyreumatic substance, and the extract.

2. The effects produced are very transitory, the poison finding a ready exit from the body.

3. All the evils of smoking are functional in character; and no confirmed smoker can ever be said, so long as he indulges in the habit, to be well. But it does not follow that he is becoming the subject of organic and fatal disease because he smokes!

4. Smoking produces disturbances in the blood, of the stomach, of the heart, of the organs of sense, of the brain, of the nervous filaments and sympathetic or organic nerves, of the mucous membrane of the mouth, and of the bronchial surface of the lungs.

5. The statements to the effect that tobacco-smoke causes specific diseases—such as insanity, epilepsy, St. Vitus' dance, apoplexy, organic disease of the heart, cancer and consumption—have been made without any sufficient evidence or reference to facts. All such statements are devoid of truth, and can never accomplish the object which those who propose them have in view.

6. As the human body is maintained alive and in full vigor by its capacity within certain well-defined limits to absorb and apply oxygen, as the process of oxydation is most *active and most required* in those periods of life when the structures of the body are attaining their full development, and as tobacco-smoke possesses the power of arresting such oxydation, the habit of smoking is deleterious to the young.

7. In the main, smoking is a luxury which any nation of moral habits would be better without. The luxury is not directly fatal to life, but its use conveys to the mind of the man who looks upon it calmly, the unmistakable idea of physical detriment.

8. But as a luxury, tending to this condition, it is probably one of the least hurtful of luxuries. It is on this ground, in fact, that tobacco holds so firm a position: that of nearly every luxury it is the least injurious. It is inoccuous as compared with alcohol; it does infinitely less harm than sugar (?); it is in no sense worse than tea; and, by the side of high living, altogether it contrasts most favorably. It is most antidotal to gluttony.

9. Tobacco may also be considered, in certain cases, as a remedy for evils that lie deeper than its own, and as such a remedy, it will persist in holding its place until those evils be removed.

*Hydrophobia Eleven Months after the Bite of a Rabid Dog.*—M. Dupuy, of Lyons, lately mentioned before the Society of Medical Sciences of that city, the case of a young lady, sixteen years of age, who was bitten by a dog which had given signs of hydrophobia. The patient had with her a favorite dog, which was attacked by the rabid animal, and whilst trying to save her own dog, the girl was severely bitten in the hand. The wounds, which bled rather freely, were not regularly dressed, and soon cicatrized. Some suspicions were afterwards entertained, and the girl had, probably from fear, hysterical attacks, which M. Dupy states were not rabid fits. A journey was then undertaken, and all fears soon vanished, until eleven months had elapsed since the accident. At that period the young lady was seized with unmistakable signs of hydrophobia, and died in a few days.

*Calabar Bean as a Neutralizer of Atropine.*—A thorough and satisfactory examination of the retina and choroid, such as is often needed for a proper knowledge and conscientious treatment of diseases of the internal tissues and humorus of the eyeball, cannot be effected without dilatlng the pupil, so as to increase the number of rays which enter and illuminate the eyeball, and to enlarge the field of observation. If the pupil be left dilated and the accommodation effected by atropine, as has hitherto been inevitably the case after its employment for ophthalmoscopic purposes, the patient suffers considerable inconvenience for a time from the inequality of the visual powers of the two eyes. Hence, too, he is often led to believe that the surgeon has inflicted some actual injury, and permanently damaged his sight, by the harmless process of dilatation; and many a patient suffering from progressive amblyopia, absurdly enough, yet from a comprehensible error, ascribes the date of visible progress of his disease to the date of the temporary dilatation of the pupil, with its attendant obvious inconveniencies. It is desirable to avoid misconceptions in practice, especially when patients are not sufficiently intelligent to understand the harmless nature of the temporary dilatation of the pupil by a drop of a weak solution of atropine. It had very early been obvious that the Calabar bean might probably furnish an active principle which might be safely and innocently employed to counteract the dilatation produced by atropine. Mr. Ernest Hart employed the beans experimentally for this purpose. It was at once obvious, from the first series of observations, that the bean fully possessed the power ascribed to it; but the best mode of employing it, the means of adjusting its application so as not to carry the effects too far, could not at once be determined. The most convenient method, for ordinary purposes, is by saturating with the extract thin paper, so adjusting the strength of the solution that equal portions of the Calabar bean paper and atropine paper might be made to neutralize each other, and leave the eye, after ophthalmoscopic examination, as nearly as possible in *statu quo.* The nearest approach to this object is attained by paper prepared according to the following formula: One ounce of the white portion of the bean is exhausted by two ounces of rectified spirit; the solution is now evaporated to one-eighth of its bulk, and then the paper is soaked in it and dried. We may add, in reference to atropine paper, that it is so prepared in London that a piece one-fifth of an inch square contains as much atropine as one drop of a two grain solution to the ounce.

*Investigations Touching the Use of Iodine.*—Dr. Rosenthal, assistant physician at the Vienna General Hospital, has published, in the *Weiner Med. Wochensch*, a series of papers containing much original matter touching the therapeutic use of iodine. The summing up is as follows:

1. Large doses of iodide of potassium, combined with a small quantity of fluid, remain a long time in the economy; with large quantities of fluid, they are quickly washed away from the system, and pass rapidly into the secretions and excretions. This circumstance should be carefully noticed.

2. When iodide potassium is taken internally, it is found, not only in the urine, saliva, and other secretions, but also in the alvine evacuations, within from four to seven hours, whether the stools be aqueous or the reverse.

3. In the administration of iodide of iron, iodine is separated in considerable quantities and found with a large proportion of the iron in the urine. Fæcal matter contains much iron and a small amount of iodine. The same phenomena may be noticed when iodide of mercury is used.

4. Frictions with an ointment containing iodide of potassium upon sound skin will cause the iodine to be detected in the urine and saliva.

5. Iodine is found in the urine of those who take baths in which iodide of potassium is dissolved, even when the rectum and urethra are kept free from the action of the bath. This is proved by examining the urine, and by noticing a large diminution of the iodine in the water used for the bath.

6. The intestinal mucous membrane takes up the iodine very energetically in the form of enemata, and this is the case even with very weak solutions of iodide of potassium.

7. Large doses of iodide of potassium, or small doses taken for a long time, are not well borne in certain pathological states of the economy; in fact large doses of iodine, or concentrated solutions, are very prejudicial to the system.

### *Whooping-Cough treated with Bromide of Ammonium.*

Dr. Harley says of this remedy:—"It must still be fresh in the memory of our readers, that Dr. Gibb discovered in the bromide of ammonium a most valuable pharyngeal and laryngeal anæsthetic;" and it is this special character of the remedy which Dr. Harley has endeavored to turn to useful practical account in the treatment of pertussis. He remarks—"During the last six months a very great number of children have suffered from whooping-cough; and every general practitioner, as well as hospital physician, knows that the success in the treatment of this troublesome affection has not at all been in proportion to our experience of the disease. Indeed, there are few diseases the diagnosis of which is so easy, the pathology so obscure, and the treatment so uncertain, as those of common whooping-cough. Specifics in abundance have been at various times proposed; but one after another, after a year or two's trial, has gradually fallen into disrepute. Although the pathology of whooping-cough is still very obscure, one thing is evident—namely, that the exciting cause of the whoop is the reflex irritation of the branches of the pneumogastric nerves. The pneumogastric nerves supply the glottis by means of their recurrent, the lungs by their pulmonary, the stomach by their gastric, and the diaphragm by their diaphragmatic branches; and let the nerve irritation originate where it may, one thing at least is clear—namely, that the immediate result is spasmodic action of all the parts supplied by the vagi. Thus it is we have the violent expulsive cough, followed by the spasmodic constriction of the glottis, impeding the free return of air to the lungs, and thereby producing the peculiar sound from which the disease takes its name. Next we have the spasmodic action of the stomach, inducing vomiting, and that again is aided by the convulsive contractions of the diaphragmatic muscles." Such being Dr. Harley's views, his object in giving bromide of ammonium was to induce, if not semi-paralysis, at least partial insensibility, of the glottis, and thereby, if possible, prevent the occurrence of the spasm, which is undoubtedly the chief source of misery during the attack. With regard to the dose: for infants, two or three grains three times a day are enough; to older children, from four to eight grains may be given, and in some cases, where the symptoms are remarkably severe, even ten grains. The simpler the vehicle the better, but if there is a tendency to bronchial or pneumonic inflammation it should be combined with either a mixture or the wine of ipecac. The special nervous symptoms seem to be more under the special control of the drug than the catarrhal, for the spasms diminish in frequency and severity, and consequently the whoop is not so often heard, showing a subsidence of the active symptoms. Dr. Gibb says that, *pari passu*, the cure is not more speedy than from the dilute nitric acid in uncomplicated cases; nevertheless, it is worthy of a more extended trial, especially in severe and obstinate cases.

*Case of Copper Colic—Third Attack; Purple Line around the Gums; Recovery.*—As it is only a few years since cases of copper colic attracted the attention of medical men, and as they are still, comparatively speaking, rare, the following instance will be read with interest. Charles S——, aged eighteen, a copper-plate printer, was admitted on the third of July into University College Hospital, complaining of severe colic. He stated that while sitting reading on the previous evening, he was suddenly attacked with acute pain in the abdomen; he felt "just as if some one had struck him violently in the belly." The pain, which he described as "a dead pain, increasing every now and then," lasted fourteen hours. The pain was not relieved on pressure; on the contrary, it was increased by it. He had no diarrhœa, nor was he costive; the bowels were moved once daily. He said that he felt very sick. Had a peculiar sallow, almost clay-colored complexion, and an anxious expression. The lips were livid, and the eyes sunken. The tongue was tolerably clean, but round the gums there was the characteristic purple line of copper poisoning. The line had not the slate-grey hue of lead, nor was there any constipation as in lead colic. On inquiring into his history it appeared that he had followed the occupation of a copper-plate printer for the last two years. He cleans the plates with a leather, and latterly he has had to clean some old copper-plates which had lain by for a long time. While cleaning them he found "a good deal of greenish dust rose like verdigris," which he had to inhale. It also appeared that he had had two similar attacks before this—the first in October, which lasted a fortnight, the other a month ago, which lasted only three days. In order to induce the elimination of the poison from the system as rapidly as possible, the following medicine was ordered to be taken three times a day:—Spirits of nitric ether, a drachm; dilute sulphuric acid, ten minims; sulphate of magnesia, a drachm; laudanum, six minims, in camphor water. Under the use of this remedy he was dismissed cured in seven days, but with the line on the gums still distinct.—*Lancet.*

*Death-Rate in Small Pox.*—Mr. Marson, the resident surgeon of the Small Pox and Vaccination Hospital at Highgate, states that of unvaccinated persons who are attacked 36 per cent. die, whilst of those attacked after vaccination the mortality is 6.76 per cent., or 1 in 15. And, further, that if after the operation there remains permanently on the arm but one scar, the mortality is 7.57 per cent.; if two scars, 4.13 per cent.; if three, 1.85 per cent.; and if four scars remain the mortality is reduced to 0.74 per cent. Facts like these, backed by the experience of a man like Mr. Marson, ought surely be sufficient to silence, if not convince, the rabid opponents of vaccination. We should have been glad to learn from a gentleman of the unusual experience of Mr. Marson the influence which revaccination exercises upon the occurrence and fatality of small pox. What is the real influence of secondary vaccination? At what period should it be performed? Is it necessary immediately after puberty or at the termination of every seventh year? These are questions of vital import, and no doubt Mr. Marson will feel it his duty to give an answer to them. Without anticipating his reply, we may state that in a practice of no inconsiderable extent not a single case of small pox has occurred after revaccination. There is no question in which the public are interested that has a more important bearing upon the general welfare than this. There is none, probably, upon which there is a greater diversity of opinion. The records of the small pox hospital, we think, ought to afford something like a satisfactory answer. It would be greatly conducive to a settlement of this question if Mr. Marson would state his experience upon this point. No man is more competent by his long experience, and by the unbiased judgment which he can give upon such a matter, to relieve the anxiety of the public upon a disputed question than the highly respected resident surgeon of the Small Pox and Vaccination Hospital.—*Lancet.*

*Artificial Formation of Fibrin.*—In a paper read by Mr. A. Smee, before the Royal Society, he showed that if a stream of oxygen gas be passed through albumen, derived from the serum of the blood, or eggs, or gluten of wheat, portions of it become converted into fibrin. If, however, a small quantity of potash be introduced, fibrin is not formed. It has been thought that the discovery may throw some light on the phenomena of fibrinous diseases, as phthisis and peritonitis, andon the use of potash in their treatment. Dr. Shettle states that albumen can be readily converted into beautiful specimens of fibrin. He immersed some zinc and copper plates in pure albumen, obtained from the white of egg, and also some in a solution of albumen, in distilled water, such solution previous to insertion of plates having been filtered through a double fold of blotting paper. In both instances most satisfactory results were obtained, the albumen coagulating on the zincode, and under the microscope, exhibiting perfect specimens of fibrin, as well as granular cells. These experiments were made from a belief previously entertained that the coagulation of blood was due to electric action—a belief strengthened by the fact that the author had previously detected evidences of electricity in arterial blood by means of DuBois Raymond's galvanometer. He is therefore disposed to believe the theory, that the conversion of albumen into fibrin is not due to the direct effect of oxygen gas, but to electric action, set up by the union of oxygen with some one or other of the proximate principles of albumen; and this view, he considers, is confirmed by the opinion of Dr. Ireland on the "Influences of Ozonized Air on Animals." Dr. Shettle has been conducting other experiments on the same principle, by acting on a dilute solution of the sap of various kinds of vegetable structure, which also, under the electric influence, has been converted into perfect specimens of tissue, in every respect resembling the structure of the plant from which the sap was taken.

*Iridectomy.*—Iridectomy is an operation for the removal or cutting away of a portion of the iris. The derivation of the term is from two Greek words. The operation is performed as follows:—The patient being placed on his back on a table or bed, the surgeon, standing at his head, makes an incision into the margin of the cornea for about one-fourth of its circumference, by entering either a Wenzel or lancet-shaped knife on its flat, and carrying the point to the opposite side, close in front of the iris, but carefully avoiding it and the lens. As the knife is withdrawn, the aqueous humour gushes out, and frequently a portion of the iris with it, through the wound, in which case it (the iris) is to be seized by forceps, and carefully cut through from its pupillary to its ciliary margin on either side of the forceps with scissors, and th portion thus cut through removed by tearing it away from its ciliary attachment. When the iris does not follow the aqueous humour through the wound, it is to be drawn out by forceps, care being taken not to injure the lens, and the operation is then completed as in the other case. The quantity of iris removed should be about one-sixth. If there be much bleeding into the anterior chamber during the operation, it will be better to remove the blood by means of a fine scoop before the eye is closed and bandages are applied.

*Phosphorous and Yellow Fever.*—In examining several of the points connected with yellow fever and its supposed causes, M. Melier calls attention to a fact which is highly interesting and suggestive—namely, that the malady more generally rages in those parts round which the phosphoresence of the sea is most prevalent. M. Melier likewise points out a fact first noticed by M. Lancereaux—namely, that the pathological lesions observed in yellow fever are exactly those of phosphoric poisoning, and that the greater part of those persons who have died from the baneful effects of phosphorous, have presented the very condition of liver considered as characteristic of yellow fever.

*Case of Transposition of the Great Vessels of the Heart.* By JOHN COCKLE, M. D.—The subject of the case was a boy, who lived to the age of two years and eight months. He was born at the full period, perfectly healthy, and remained so until the end of the third month. He now became cyanosed, and suffered from cough and dyspnœa. His lower limbs were always weak; he was also very sensitive to cold. Under emotion the cyanotic tint darkened, and the dyspnœa and habitually increased impulse of the heart would be still farther increased. No convulsions, however, occurred. The child was remarkable for unusual intelligence. Eventually he lost flesh and became dropsical and covered with petechiæ, and died apparently from exhaustion.

The heart was found dilated and hypertrophied. The aorta arose from the right ventricle, and divided into its ordinary branches. The pulmonary artery, with its valves very large, arose from the left ventricle, and normally divided. The ductus arteriosus was perfectly closed. Foramen ovale largely patent. Septum ventriculare perfect. The various orifices and valves healthy. The mitral valve, however, was, as it were, tricuspid. The left lung was in part unexpanded.

The author finds that the number of cases of transposition recorded amounts to about thirty-two. Only three cases, however, are on record, in which the foramen ovale was alone patent, the arterial duct being closed, and the ventricular septum entire. In such cases, were it not that some interchange of blood occurred through the foramen ovale, two perfectly distinct circulating systems would have existed. No other vessels certainly compensated in this case.

*Dysmenorrhœa and Sterility.*—Before the Obstetrical Society of London, Dr. Greenhalgh read a paper on the importance, frequency and close connection of these affections, and directed the attention of the Fellows to the treatment of mechanical dysmenorrhœa, which a large experience had convinced him was by far the most frequent form of this complaint. Having compared the relative merits of dilatation and division of the os and cervix uteri in this affection, he expressed a decided preference in favor of the latter mode, for the safe performance of which operation he had invented an instrument, which he proposed to call the "bilateral metrotome." He stated that he had used this instrument in upwards of thirty cases, without a single casualty, and in the great majority with the best results. After giving a description of this metrotome to the Society, together with a brief summary of the cases acted upon, and enumerating the affections in which he had found the division of the os and cervix uteri most serviceable, he pointed out the necessity of attending to the pathological states of the uterus, frequently induced by the persistence of dysmenorrhœa, and concluded by giving a short account of the plan and remedies which he had found most beneficial for the cure of these several conditions.

*Nascent Carbonate of Iron.*—Messrs. Garnier and Lamoureux, says the *Gazette des Hopitaux,* make granules of one-fifth of a grain of sulphate of iron, which are then coated with sugar. These granules are subsequently surrounded by a layer of bicarbonate of soda, and a second coating of sugar is applied. The two salts thus separated have no action upon each other as long as they are kept dry. On reaching the stomach, the granule dissolves, and carbonate of iron and sulphate of soda are formed. The gastric juice act at once upon the carbonate of iron at the very time of the formas tion of the salt, and the small amount of sulphate of soda has a tendency to counteract the constipating effect of the steel. The number of granules taken at the beginning of a meal, vary from five to thirty.

*Action of Solar Rays on Exposed Intestines.*—At a meeting of the Academy of Medicine of Paris, M. Sappey read a report on a case of severe wound of the abdomen. The patient was a shepherd boy, aged eleven, who was gored by a bull, and to such an extent that the stomach, spleen, and a large portion of the intestines were protruding. Being far from any help, the poor boy lay for two hours with the viscera just mentioned exposed to the action of the broiling sun. Dr. Patry found the patient in this pitiable state, and, by dint of care and perseverance, the boy recovered. His medical attendant seized, however, upon this opportunity, to watch the mechanism of vomiting, and found that the phenomena succeeded each other in the following manner: Contraction of the diaphragm—vermicular contraction of the stomach, commencing at the pylorus and running from the latter to the cardiac orifice, forcing the liquids contained in the stomach towards the œsophageal opening—energetic contraction of the œsophagus—involution of the stomach at every effort—dilatation of the cardia under the influence of the longitudinal fibres of the œsophagus—finally, filling of the latter canal by the liquids coming from the stomach, and vomiting.

*Discovery of a Medical Papyrus at Thebes.*—Mr. A. Leith Adams, of Malta, describes a medical papyrus discovered at Thebes, which is written in a demotic character, and contains a discourse on boils (ulcers,) fractures, and wounds on the head in general, with aphorisms, prognostics, and an oath or prayer to be used by the physician in attendance on the sick, the whole being somewhat after the style of the Hipocratic writings and the early Grecian schooן of medicine.

*Iodine, Bromine, Chlorine.*—In a note to the Academy of Sciences, M. Alexandre de la Roche draws attention to the great resemblance in the chemical behaviour of the three principles—iodine, bromine, and chlorine; and to the probability of their being compounds of a common base. All three, he remarks, have a great affinity for hydrogen, forming acids with nearly identical properties, yielding white vapours when placed in contact with air, being exceedingly soluble in water, acting in the same manner upon vegetable substances, and being generally found in combination with sodium. He likewise deems it probable that both selenium and sulphur are similarly allied.

*Jacob's Ladder.*—In his eulogium delivered on the late Geoffrey de St. Hilaire, M. Drouyn de l'Huys says: "It was undoubtedly the love of his fellows which led Geoffroy St. Helaire into the path in which we have followed him; we owe it to his memory to continue his work. May the master minds of science march at our head, and pursue through his iniative that work of ages which brings the intelligence of man in contact with the eternal truth! Science is that mysterious ladder on which Jacob saw, while dreaming, angels ascending and descending to convey heavenwards the aspirations of earth, and to convey back to earth the benedictions of Heaven." (!)

*Iodized Oil.*—M. Personne, Chief Apothecary of the Pitié, has just written a little work detailing the results of experiments undertaken with the view of testing the relative value of iodized oil and cod-liver oil in the treatment of scrofulous and other cathectic maladies. The facts are striking, and well-supported by proper names of most respectable authority. The cod henceforth will have little else to do than hide his diminished liver.

*M. Sperino's Treatment of Lenticular Cataract.*—It would appear from a letter of M. Sperino to the *Gaz. Med.* of Turin, that he has succeeded in several cases in rendering the opaque lens transparent after a certain time, by daily evacuating the *aqueous humour*. M. Sperino says that he will soon publish his cases.

*Alkalies in Rheumatism.*—Dr. Furnivall urges strongly on his medical brethren the propriety of treating *all* cases of rheumatism with alkalies, as *essential* to the prevention of cardiac sequalæ. He states that he believes there is some abnormal alteration of the blood, of acid tendency, only to be counteracted by alkalies. Dr. Wright, of Birmingham, tried the alkaline plan largely in rheumatism, and gave us a very favorable account of the results. Since then the agency of lactic acid in causing rheumatic cardiac disease and deposits has been proved.

